# HDAC6 Regulates the Fusion of Autophagosome and Lysosome to Involve in Odontoblast Differentiation

**DOI:** 10.3389/fcell.2020.605609

**Published:** 2020-11-26

**Authors:** Yunyan Zhan, Haisheng Wang, Lu Zhang, Fei Pei, Zhi Chen

**Affiliations:** The State Key Laboratory Breeding Base of Basic Science of Stomatology (Hubei-MOST) and Key Laboratory for Oral Biomedicine of Ministry of Education (KLOBM), School and Hospital of Stomatology, Wuhan University, Wuhan, China

**Keywords:** differentiation, odontoblast, lysosome, autophagosome, fusion, HDAC6

## Abstract

Odontoblast differentiation is an important process during tooth development in which pre-odontoblasts undergo elongation, polarization, and finally become mature secretory odontoblasts. Many factors have been found to regulate the process, and our previous studies demonstrated that autophagy plays an important role in tooth development and promotes odontoblastic differentiation in an inflammatory environment. However, it remains unclear how autophagy is modulated during odontoblast differentiation. In this study, we found that HDAC6 was involved in odontoblast differentiation. The odontoblastic differentiation capacity of human dental papilla cells was impaired upon HDAC6 inhibition. Moreover, we found that HDAC6 and autophagy exhibited similar expression patterns during odontoblast differentiation both *in vivo* and *in vitro*; the expression of HDAC6 and the autophagy related proteins ATG5 and LC3 increased as differentiation progressed. Upon knockdown of HDAC6, LC3 puncta were increased in cytoplasm and the autophagy substrate P62 was also increased, suggesting that autophagic flux was affected in human dental papilla cells. Next, we determined the mechanism during odontoblastic differentiation and found that the HDAC6 substrate acetylated-Tubulin was up-regulated when HDAC6 was knocked down, and LAMP2, LC3, and P62 protein levels were increased; however, the levels of ATG5 and Beclin1 showed no obvious change. Autophagosomes accumulated while the number of autolysosomes was decreased as determined by mRFP-GFP-LC3 plasmid labeling. This suggested that the fusion between autophagosomes and lysosomes was blocked, thus affecting the autophagic process during odontoblast differentiation. In conclusion, HDAC6 regulates the fusion of autophagosomes and lysosomes during odontoblast differentiation. When HDAC6 is inhibited, autophagosomes can't fuse with lysosomes, autophagy activity is decreased, and it leads to down-regulation of odontoblastic differentiation capacity. This provides a new perspective on the role of autophagy in odontoblast differentiation.

## Introduction

Odontogenesis is a complex process involving reciprocal interactions between the dental epithelium and mesenchymal cells (Thesleff and Nieminen, 1996). During this process, odontoblasts derived from cranial neural crest-originating mesenchyme cells produce dentin, which makes up the main portion of the tooth (Ruch et al., 1995; Kawashima and Okiji, 2016). Odontoblasts are a layer of long-living post-mitotic dental cells aligned in an ordered manner along the dental pulp (Sasaki and Garant, 1996). During odontoblast differentiation, pre-odontoblasts exit the cell cycle and then undergo cell growth, elongation, and polarization to finally become highly complex polarized secretory odontoblasts (Zhang et al., 2005; Biz et al., 2010). One major function of odontoblasts is to synthesize and secrete predentin matrix components, such as collagen type I, proteoglycans, and non-collagenous proteins like dentin sialoprotein (DSP) (Ruch et al., 1995; Sasaki and Garant, 1996). Thus, there are many activities, such as protein synthesis, protein turnover, and substance transport, taking place in odontoblasts.

Autophagy is an evolutionarily conserved homeostatic process involved in the degradation and recycling of proteins to maintain cell homeostasis and supply energy (He and Klionsky, 2009). Autophagy is initiated by the formation of double membrane vesicles that encapsulate cargo proteins or organelles to form mature autophagosomes (Hale et al., 2013). The autophagosomes go on to fuse with lysosomes to form autophagolysosomes and degrade their cargo (Zhang et al., 2013). As a recycling system, autophagy plays an important role in a variety of physiological process such as cell differentiation (Salemi et al., 2012), cell pluripotency (Sharif et al., 2017), and cell death (Denton et al., 2015). Recently, many studies have shown that autophagy is involved in various physiological process of odontoblasts. For example, the specific spatiotemporal expression pattern of LC3 in the tooth germ indicates that autophagy is involved in tooth development (Yang et al., 2013). Additionally, the autophagic-lysosomal system is involved in odontoblast longevity and aging (Couve et al., 2013). Autophagy is also thought to play a dual role in inflamed odontoblasts by modulating odontoblastic differentiation in the inflammatory microenvironment (Pei et al., 2016). However, the molecular mechanism by which autophagy regulates odontoblast differentiation remains largely unknown.

Histone deacetylases (HDACs) are a group of enzymes that remove acetyl groups from both histone and non-histone proteins (Gregoretti et al., 2004). HDAC6 is a unique member of the type II HDACs that is primarily localized in the cytoplasm; it contains two functional catalytic domains and a ubiquitin-binding zinc finger domain for the regulation of ubiquitination-mediated degradation (Hard et al., 2010). In addition to histones, the substrates of HDAC6 include acetylated-Tubulin (ac-Tubulin), Cortactin, retinoic acid inducible gene I (RIG-1), Hsp90, and β-Catenin (Zhang et al., 2015). Given its unique molecular structure and the diversity of its substrates, HDAC6 plays a vital role in cell migration, the immune response, and cell proliferation and differentiation (Hai et al., 2011; Li et al., 2013; Zheng et al., 2017). Recently, it has been found that HDAC activity plays a crucial role in osteoblast maturation (Ehnert et al., 2012). HDAC1 and HDAC3, which are type I HDACs, are thought to inhibit Runx2 expression and affect osteoblast differentiation (Schroeder et al., 2004; Liu et al., 2015). HDAC3 has been shown to interact with KLF4 during the initiation of odontoblastic induction (Tao et al., 2019). HDAC6 is also involved in human osteoblast mechanosensation (Ehnert et al., 2017). However, there are few studies of HDAC6 in odontoblasts, and the role of HDAC6 in odontoblast differentiation remains unclear.

Moreover, it has been found that autophagy and HDAC6 are closely related in many conditions. For example, in human breast cancer, HDAC6 forms complexes with CDK1 and induces its P62-dependent autophagy degradation (Galindo-Moreno et al., 2017). In pancreatic cancer, MicroRNA-221 induces autophagy through suppressing HDAC6 expression (Yang et al., 2018). During viral infection, HDAC6 induces stress granule autophagic degradation by acting as a cargo that is recognized by P62 (Zheng et al., 2020a). In Parkinson's disease, HDAC6 promotes autophagosome-lysosome fusion and autophagy (Wang et al., 2019). This indicates that HDAC6 can either induce or reduce autophagy in different pathologies. However, whether and how HDAC6 regulates autophagy in odontoblasts is unclear.

In this study, to further investigate the role of autophagy in odontoblast differentiation, we explored the role of HDAC6 in odontoblast differentiation and the relationship of HDAC6 and autophagy during this process.

## Materials and Methods

### Cell Isolation and Culture

Human dental papilla cells (hDPCs) were isolated from the dental papilla tissues of the tooth germ of human third molars (Sonoyama et al., 2006). Dental papilla tissues were obtained from extracted lower third molar germ of healthy donors (12 years old) with informed consent. All procedures were performed according to the guidelines of the National Institutes of Health in regard to the use of human tissues and with permission by the Ethics Committee of the School and Hospital of Stomatology, Wuhan University (protocol No. 2018A62). After extraction, the tissue was washed three times with phosphate-buffered saline (PBS, HyClone, Logan, UT, United States) containing 2% penicillin and streptomycin (HyClone). Then, the dental papilla tissues were cut into small pieces (~1 mm^3^ in size). The tissue pieces were seeded into a T25 cell culture flask in α-modified Eagle's medium (α-MEM, HyClone) with 20% fetal bovine serum (FBS, Gibco, Thornton, NSW, Australia) and 1% penicillin and streptomycin (HyClone). The growth medium was changed once a week until the cells grew out, and then it was changed twice a week. Upon reaching confluence, cells were passaged at a 3-fold dilution. When the cells were passaged, the culture medium changed to include 10% fetal bovine serum. Cells between passage 3 and 6 were used in this study.

### *In vitro* Odontoblastic Differentiation and Tubacin Treatment

For odontoblastic induction, cells were cultured as described previously (Chen et al., 2005; Pei et al., 2016). hDPCs were seeded in 6-well plates at a density of 2 × 10^5^ cells per well and cultured in mineralization-inducing medium (MM), which was α-MEM with 10% FBS, 1% penicillin and streptomycin, 10 mM sodium β-glycerophosphate (Sigma, St Louis, MO, USA), 50 mg/ml ascorbic acid (Sigma) and 10 nM dexamethasone (Sigma). MM was changed every 2 days. Cells were harvested 2 weeks after induction and underwent Alizarin Red-S staining to analyze calcified nodule formation. Proteins were harvested at different time points for western blots and were extracted at 2 weeks for alkaline phosphatase activity assays.

To inhibit the HDAC6 activity, Tubacin (Item No. 13691, Cayman Chemical Company, Michigan, United States) was added in the culture medium in a final concentration of 7.5 μmol/L of the cells with or without MM (Haggarty et al., 2003).

### Small Interfering RNA and mRFP-GFP-LC3 Plasmid Transfection

For functional knock-down of HDAC6, an *HDAC6*-targeting small interfering RNA (siRNA; *siHDAC6*, Sigma) and the universal negative control siRNA were transfected into hDPCs. Cells were seeded into 6-well plates and then transfected with siRNA using Lipo2000 (Invitrogen, Thermo Fisher Scientific, Waltham, MA, United States) according to the manufacturer's instructions (Tao et al., 2019). Knockdown efficacy was confirmed by western blot. Odontoblastic differentiation was induced 48 h after transfection. For the tracing of autophagic flux, mRFP-GFP-LC3 plasmid (plasmid 21074; Addgene) was transfected into hDPCs following transfection with siRNA prior to the induction of odontoblastic differentiation using Lipo2000 according to the manufacturer's instructions(Kimura et al., 2007).

### Double and Triple Immunofluorescence Staining

Double and triple immunofluorescence staining was performed as previously described (Pei et al., 2016). The lower mandibles of postnatal day 2.5 mice were dissected and fixed in 4% buffered paraformaldehyde, rinsed overnight, demineralized in 10% EDTA for 1 week, and then embedded in paraffin after being dehydrated. The paraffin block–embedded tissue was cut into 5 μm sections. After deparaffinizing, rehydrating, and undergoing antigen retrieval using a microwave, the tissues were blocked with 2.5% bovine serum albumin (BSA, Sigma–Aldrich, St Louis, MO, United States) for 1 h at 37°C, then incubated with anti-HDAC6 (1:100, Proteintech, Chicago, IL, USA) overnight at 4°C, and then incubated with Alexa Fluor 594 conjugated secondary antibody (CWBIO, Beijing, China) for 1 h. Next, the slides were again blocked with 2.5% BSA. Slides were incubated for 1 h at 37°C with primary antibodies against DSP (1:100, Santa Cruz, Biotechnology, Inc., Dallas, TX, USA) and ATG5 (1:100, Proteintech) were, followed by incubation with Alexa Fluor 488 conjugated secondary antibody following counterstaining with 4′,6-diamidino-2-phenylindole (DAPI, ZSGB-BIO, Beijing, China).

Cells were seeded on coverslips and transfected with *siHDAC6* after attachment. After inducing odontoblastic differentiation, the coverslips were washed with PBS and fixed with 4% paraformaldehyde or methyl alcohol at room temperature for 15 min. After being washed three times with PBS, the coverslips were blocked with 2.5% BSA for 1 h at 37°C, and then incubated with anti-HDAC6 primary antibody for double staining and both anti-ac-Tubulin (1:100, Proteintech) and anti-LC3 (1:100, Medical & Biological LaboratoriesCo., MBL, Nagoya, Japan) primary antibodies simultaneously for triple staining at 4°C overnight. Coverslips were then incubated with secondary antibodies conjugated with Alexa Fluor 488 or both Alexa Fluor 488 and Cy3 for 1 h. Next, the coverslips were again blocked with 2.5% BSA. After that the second primary antibodies against LC3 and HDAC6 were incubated for 1 h at 37°C, and coverslips were then incubated with Cy3- or Alexa Fluor 674-conjugated secondary antibodies, followed by counterstaining with 4′,6-diamidino-2-phenylindole (DAPI).

Tissues sections and cells were observed and photographed using a fluorescence microscope (Leica, Wetzlar, Germany) or by confocal fluorescence microscopy (Leica, Solms, Germany).

For mRFP-GFP-LC3 observation, cells were seeded on coverslips and transfected with *siHDAC6* and mRFP-GFP-LC3 plasmid after attachment. Following the induction of odontoblastic differentiation, coverslips were washed in PBS and fixed with 4% paraformaldehyde for 15 min at room temperature. After being washed three times with PBS, cells were stained with 4′,6-diamidino-2-phenylindole (DAPI) and were then directly observed by confocal fluorescence microscopy (Leica, Solms, Germany). The intensity of single red fluorescence (that not colocalized with green) and yellow fluorescence was quantified by Image Pro Plus (Media Cybernetics, Rockville, USA). The intensity was average of four random areas.

### Western Blot Analysis

Cells were harvested at different time points and lysed in lysis buffer with protease and phosphatase inhibitor on ice for 3 min. Protein supernatants were harvested following centrifugation at 13,000 rpm for 10 min. Protein concentration was measured and standardized using the BCA Protein Assay Kit (Pierce Biotechnology, Rockford, IL, USA). Twenty-five microgram total protein samples were loaded and separated by 8 or 12% sodium dodecyl sulfate–polyacrylamide gel electrophoresis and then transferred to polyvinylidene fluoride membranes (Merck Millipore, Darmstadt, Germany). Membranes were immediately blocked with 5% nonfat milk for 1 h at room temperature and then incubated with the following primary antibodies: anti-HDAC6 (1:1,000, Proteintech), anti-DMP1 (1:4,000, Abcam, Cambridge, MA, USA), anti-DSP (1:1,000, Santa Cruz), anti-OSX (1:1,000, Santa Cruz), anti-GAPDH (1:8,000, Proteintech), anti-Beclin1 (1:1,000, Proteintech), anti-ATG5 (1:5,000, Abam), anti-P62 (1:1,000, Proteintech), anti-LAMP2 (1:1,000, GeneTex, Irvine, California, USA), anti-LC3 (1:1,000, MBL), anti-ac-Tubulin (1:1,000, Proteintech), and anti-Tubulin (1:1,000, Antgene, Wuhan, China) overnight at 4°C. Next, membranes were incubated with goat anti–rabbit or goat anti–mouse peroxidase–conjugated secondary antibody for 1 h at room temperature. Relative protein levels were determined by densitometry quantification using ImageJ (National Institutes of Health, Bethesda, MD, USA), and GAPDH was used as the loading control.

### Alizarin Red Staining

Alizarin red staining was performed as previously described (Pei et al., 2016). Briefly, after hDPCs were cultured in MM for 14 days, the cells were fixed with 95% ethanol for 10 min after being washed with PBS. Cells were then stained with 1% Alizarin red (Sigma-Aldrich) solution for 20 min at room temperature. Then, cells were washed to remove residual Alizarin red and were photographed with an inverted phase contrast microscope (OLYMPUS IX41, Olympus, Shinjuku-ku, Tokyo, Japan) and the mineral alizarin red staining(AR-S) from each well was quantified. Stained cells were incubated in cetylpyridinium chloride (CPC, Solarbio Science Technology Co, Beijing, China) buffer (10% w/v) in 10 mM Na_2_HPO_4_ (PH7) at 37°C for 12 h to extract the mineralized tissue and then the OD_550_nm was measured using a microplate reader. Mineralized nodule formation was represented as nmol AR-S per μg of total cellular protein. Total cellular protein content was determined by the BCA Protein Assay Kit. Two samples were used for each condition and the experiments were repeated three times.

### Alkaline Phosphatase (ALPase) Activity Assay

Alkaline phosphatase is an enzyme produced by cells and is a marker of osteoblastic/odontogenic differentiation. Alkaline phosphatase enzyme activity of the cells in different groups was measured using the Alkaline Phosphatase Assay kit (Cat. A059-2 Jiancheng Bioengineering Institute, Nanjing, China) according to the manufacturer's instructions. The ALP substrate, the disodium phenyl phosphate, can be decomposed by the alkaline phosphatase in cell lysate and produce free phenol, free phenol can be further oxidized to red quinone derivatives which represent the amount of enzyme in cell lysate (Datta et al., 2005). Briefly, the cell lysate was added to a transparent 96-well plate with 100 μl ALP substrate solution and the plate was incubated for ~15 min at 37°C in the dark. After incubation, 150 μl color development agent was added and the absorbance was measured at 520 nm using a multi-well plate reader. ALP activity was normalized to protein concentration (measured by the BCA Protein Assay Kit). Samples were run in triplicate and compared against phenol standards. ALP activity was calculated as 1 mol of phenol per mg protein.

### Statistical Analysis

All data are presented as the mean ± SEM. GraphPad Prism 5.0 (GraphPad Software, La Jolla, CA, USA) was used to analyze and visualize the data. One-way ANOVA followed by Tukey's Multiple Comparison Test was used to analyze ALP activity and Alizarin red staining quantification in the MM, MM + siNC, MM + siHDAC6, MM + Tubacin treated hDPC groups compared to the control group. Experiments were all repeated independently three times. *P* < 0.05 was considered to be statistically significant.

## Results

### HDAC6 Expression Increases During Odontoblast Differentiation

**Figure 1:**
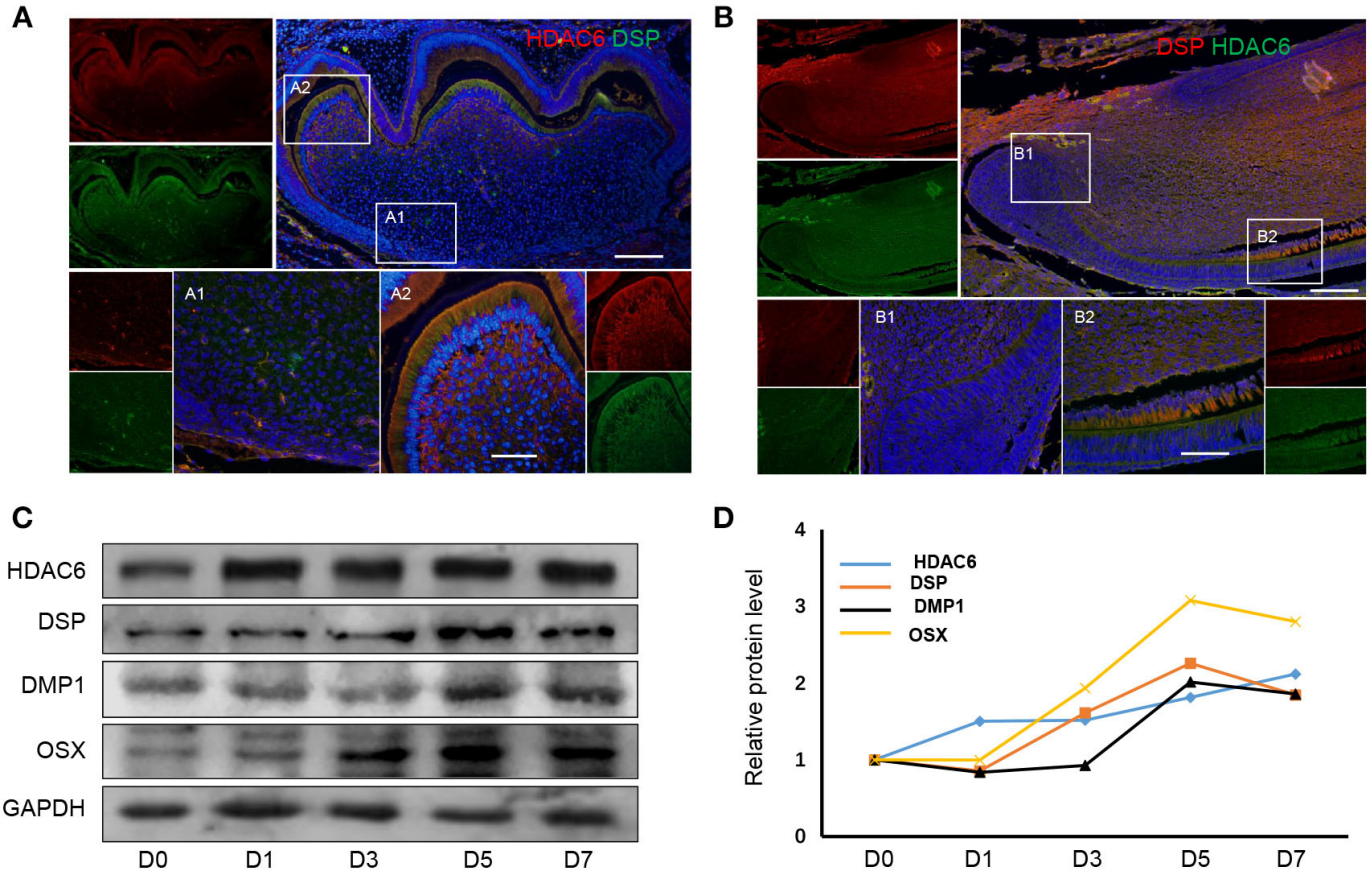
HDAC6 expression increases during odontoblast differentiation. HDAC6 expression during odontoblast differentiation *in vivo* of mouse molar **(A)** and incisor **(B)** models as detected by HDAC6 and DSP double immunofluorescence staining. DAPI counterstaining (blue) indicates nuclei, green fluorescence represents DSP **(A)** or HDAC6 **(B)**, and red fluorescence represents HDAC6 **(A)** or DSP **(B)**. Scale bars = 25 μm. **(C,D)** The protein expression levels of HDAC6, DSP, DMP1, and OSX in hDPCs were analyzed following mineralized induction for 0–7 days.

To observe the expression of HDAC6 during odontoblast differentiation, we first detected the protein expression pattern of HDAC6 during odontoblast differentiation in mouse molars and incisors. In mouse molars, HDAC6 was highly expressed in differentiated odontoblasts (Figure 1A2) when compared to undifferentiated cells in the apical region (Figure 1A1). HDAC6 colocalized with DSP in differentiated odontoblasts (Figure 1A2). In mouse incisors, which show the odontoblast differentiation process, HDAC6 exhibited a similar expression pattern. HDAC6 and the odontoblast differentiation maker DSP showed intense expression in differentiated odontoblasts (Figure 1B). Meanwhile, hDPCs were cultured in MM for 7 days to achieve odontoblastic differentiation *in vitro*. The protein levels of DSP, DMP1, and OSX increased during the differentiation process, and HDAC6 was also up-regulated in a time-dependent manner during odontoblastic differentiation (Figures 1C,D). This suggested that HDAC6 is involved in odontoblast differentiation both *in vivo* and *in vitro*.

### HDAC6 Inhibition Inhibits Odontoblastic Differentiation in hDPCs

**Figure 2:**
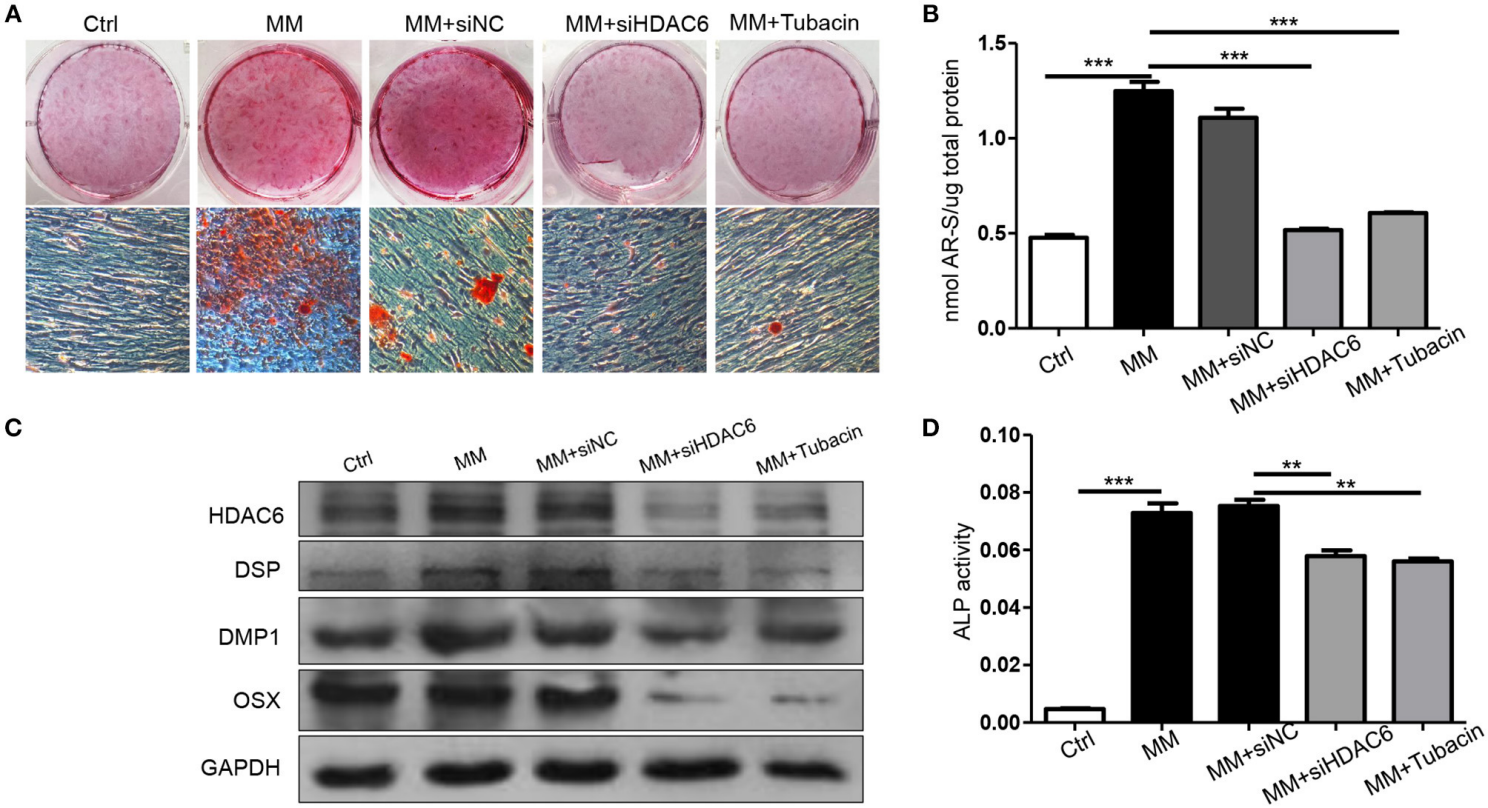
Inhibition of HDAC6 impairs the odontoblastic differentiation of hDPCs *in vitro*. hDPCs were cultured in complete medium (Ctrl), mineralization-inducing medium (MM), MM with *siHDAC6* transfection (MM + siHDAC6), and MM with the HDAC6 inhibitor Tubacin (MM + Tubacin) for 14 days. **(A)** Alizarin red S was used to stain mineralization with red in the dishes and **(B)** cetylpyridinium chloride (CPC) was used to quantitative the relative levels of Alizarin red staining. **(C)** The expression of DSP, DMP1, and OSX was examined by western blotting. **(D)** ALP activity was tested in different groups. Results are representative of three independent experiments with similar results ± SD. Data were analyzed using one-way ANOVA with Tukey's multiple comparison test. ***P < *0.001*, **P < *0.01*.

To further confirm the role of HDAC6 in odontoblast differentiation, *siHDAC6* or an inhibitor (Tubacin) was used to inhibit HDAC6 in hDPCs. Alizarin red staining revealed that the formation of mineralization nodules was clearly decreased in MM with both the *siHDAC6* (MM + *siHDAC6*) and Tubacin (MM + Tubacin) groups when compared to that of the MM group (Figure 2A). We then quantified the mineralized tissue using CPC buffer; the amount of mineralized tissue was increased in cells cultured in MM while it was significantly reduced following HDAC6 inhibition with *siHDAC6* or Tubacin (Figure 2B). Expression of the odontoblast differentiation markers DSP, DMP1, and OSX increased with MM culture, consistent with the above results. However, HDAC6 protein expression and the expression of odontoblast differentiation markers were decreased when cells were treated with *siHDAC6* or Tubacin (Figure 2C). ALP activity was also up-regulated in the MM group but was down-regulated upon HDAC6 inhibition (Figure 2D). These results demonstrated that HDAC6 played a vital role in odontoblast differentiation, and HDAC6 inhibition would inhibit the odontoblastic differentiation of hDPCs.

### HDAC6 Affects Autophagic Flux in hDPCs

**Figure 3:**
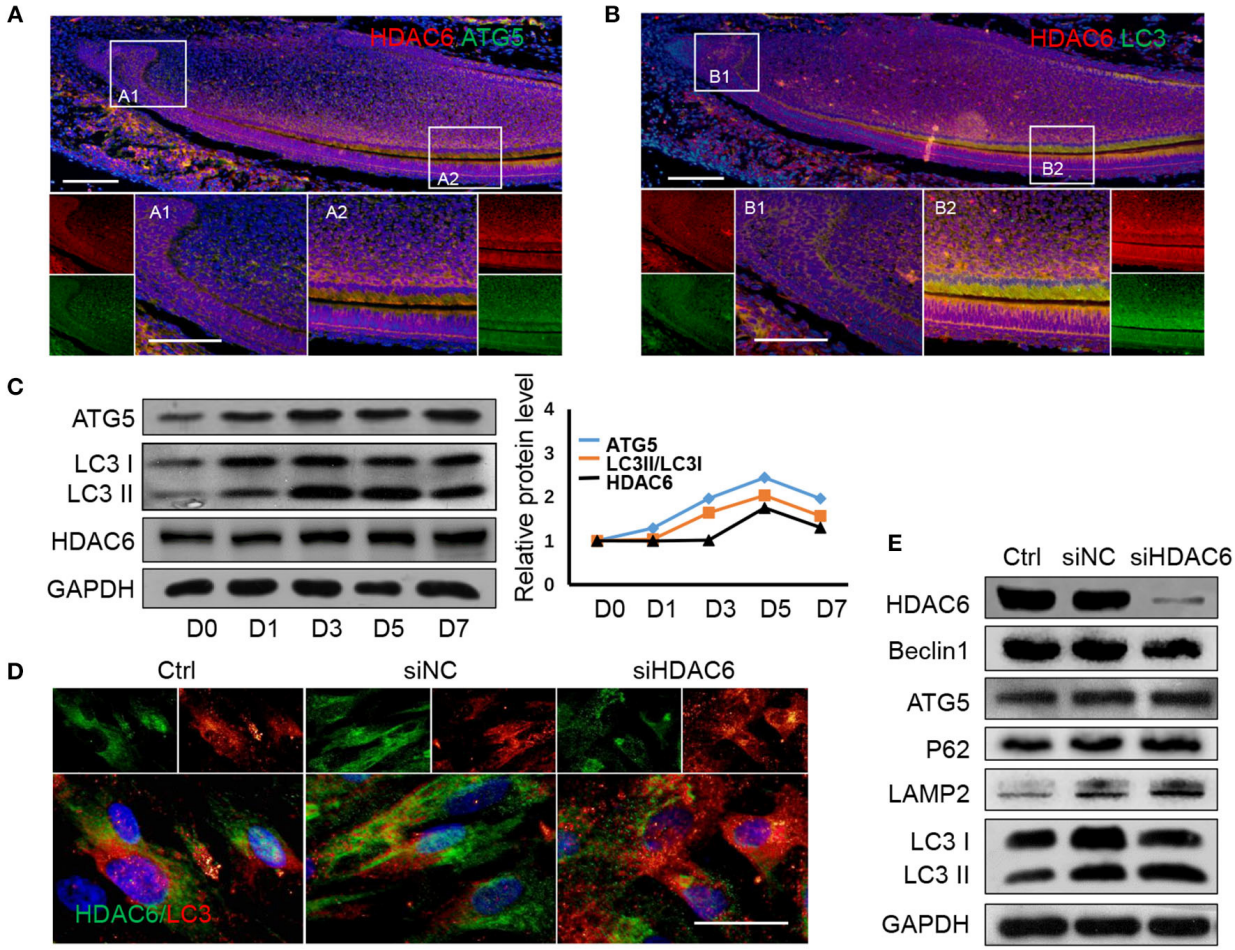
HDAC6 and autophagy related proteins show similar expression patterns during odontoblast differentiation and HDAC6 affects autophagic flux. **(A)** The expression of HDAC6 and ATG5 in mouse incisors was detected by double immunofluorescence staining. DAPI counterstaining (blue) shows nuclei, green fluorescence represents ATG5, and red fluorescence represents HDAC6. Scale bars = 25 μm. **(B)** The expression of HDAC6 and LC3 in mouse incisors were detected by double immunofluorescence staining. DAPI counterstaining (blue) shows nuclei, green fluorescence represents LC3, and red fluorescence represents HDAC6. Scale bars = 25 μm. **(C)** The protein expression levels of HDAC6 and the autophagy related molecules ATG5 and LC3II/I were analyzed following mineralized induction for 0–7 days and were quantified using densitometry (right panel). **(D)** The expression and localizatoin of HDAC6 and LC3 were detected by double immunofluorescence staining. DAPI counterstaining (blue) shows nuclei, green fluorescence represents HDAC6, and red fluorescence represents LC3. Scale bars = 50 μm. **(E)** The expression levels of HDAC6, ATG5, Beclin1, P62, and LAMP2, and the ratio of LC3II/I were examined by western blotting. Experiments were repeated in triplicate.

Given that we previously found that autophagy could regulate odontoblast differentiation for inflammatory defense (Pei et al., 2016) and in tooth development, autophagy activation was detected in differentiated odontoblasts (Yang et al., 2013). To investigate the relationship between HDAC6 and autophagy during odontoblast differentiation, we detected the expression of HDAC6 and the autophagy related molecules ATG5 and LC3 in a mouse incisor model using immunofluorescence staining. HDAC6 and ATG5 were both highly expressed in differentiated odontoblasts and were colocalized in the cytoplasm (Figure 3A). LC3 also colocalized with HDAC6 and was more highly expressed in differentiated odontoblasts than in undifferentiated cells (Figure 3B). The expression of LC3 II and ATG5 was increased during odontoblastic differentiation and peaked at 5 days (Figure 3C). While the expression of HDAC6 showed slightly change in the early time, its expression was also increased during odontoblast differentiation and peaked at 5 days (Figure 3C). These suggested HDAC6 and autophagy participated in odontoblastic differentiation and had close relationship. Next, to explore the relationship between HDAC6 and autophagy, we observed autophagic flux after knocking down HDAC6 in hDPCs. We found HDAC6 expression to be reduced after transfection with *siHDAC6*, while the signal from LC3 puncta became stronger (Figure 3D). The ratio of LC3II/LC3I was up-regulated (Figure 3E). It suggested the accumulation of autophagosomes. However, more autophagosomes don't equate with increased autophagy. A block in trafficking to lysosomes will also lead to autophagosomes accumulation, which actually indicates autophagy down-regulation (Klionsky et al., 2016). So we further detected changes in protein levels to confirm the change in autophagic flux. Interestingly, the level of the autophagy degradative substrate P62 increased following HDAC6 inhibition, suggesting that autophagy degradation was inhibited. Meanwhile Beclin1 and ATG5, which are involved in the autophagy initiation and elongation stages, showed no obvious changes, and the expression of the lysosome marker LAMP2 increased (Figure 3E). These results revealed that knocking down HDAC6 blocked autophagic flux, autophagosomes accumulated with HDAC6 inhibition in hDPCs.

### HDAC6 Deficiency Inhibits Autophagosome and Lysosome Fusion During Odontoblastic Differentiation

**Figure 4:**
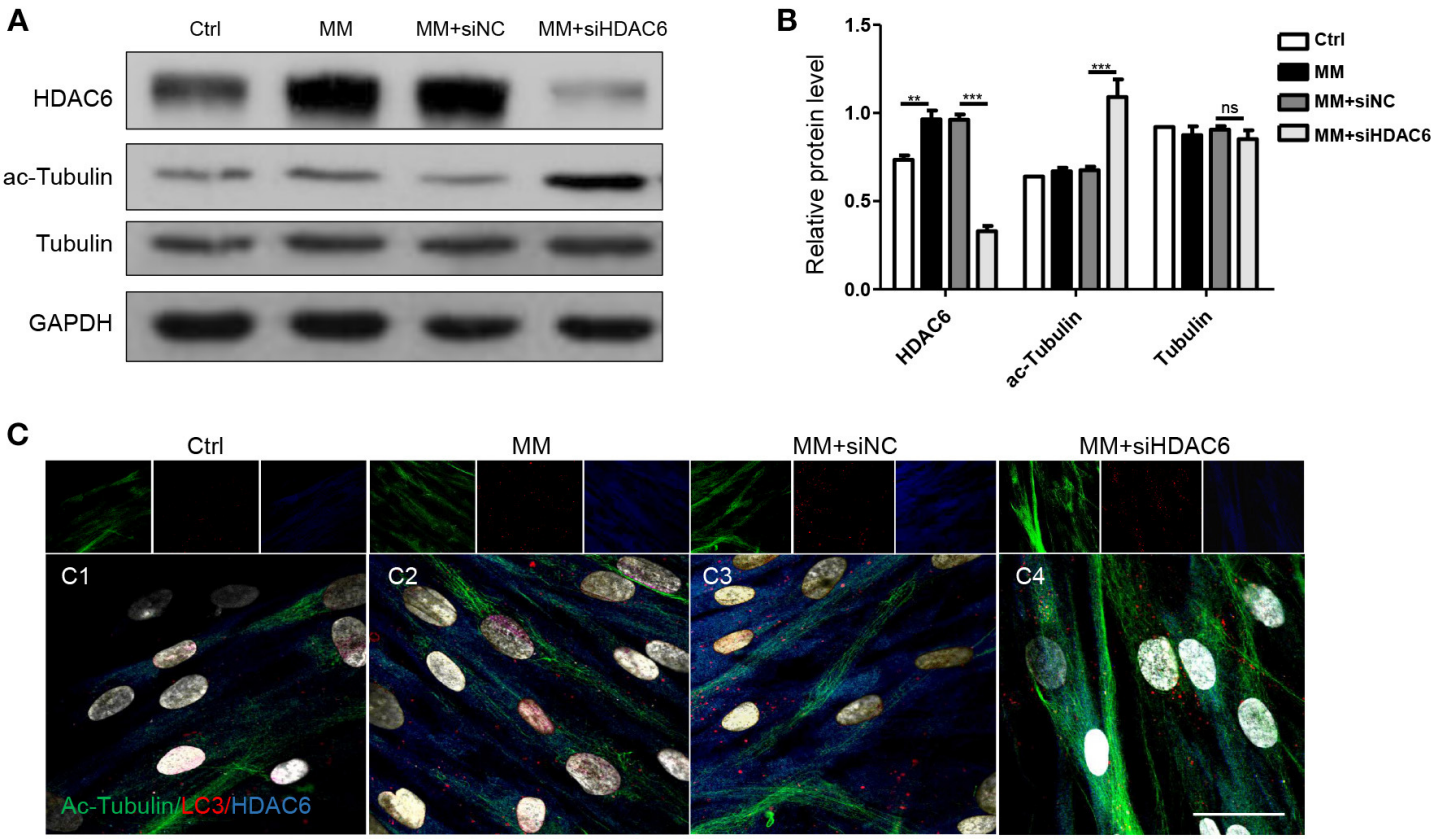
HDAC6 regulates tubulin networks during odontoblast differentiation. hDPCs were cultured in complete medium (Ctrl), mineralization-inducing medium (MM), MM following siNC (negative control) transfection, and MM following *siHDAC6* transfection. **(A)** The protein levels of HDAC6, acetylated-tubulin (ac-Tubulin), and tubulin were detected, and their relative levels **(B)** were quantified and normalized to GAPDH using Image J. Data are presented as mean ± SD. Data were analyzed using one-way ANOVA with Tukey' multiple comparison test. ***P < *0.001*, **P < *0.01*. **(C)** Triple immunofluorescence staining showed the distribution of HDAC6, ac-Tubulin, and LC3 in different conditions. DAPI counterstaining (gray) shows nuclei, green fluorescence represents ac-Tubulin, red fluorescence represents LC3, and blue fluorescence represents HDAC6. Scale bars = 25 μm.

To investigate the mechanisms by which HDAC6 regulates autophagic flux during odontoblast differentiation, we first detected the expression of the HDAC6 substrate ac-Tubulin during odontoblast differentiation. We found that the level of ac-Tubulin increased when HDAC6 was knocked down (Figures 4A,B). Next, we performed multiple immunofluorescence staining to visualize the distribution of HDAC6, ac-Tubulin, and LC3 during odontoblast differentiation. HDAC6 was distributed widely in the cytoplasm and its immunofluorescence signal became stronger upon mineralization induction (Figure 4C1,C2). Ac-Tubulin, representing the cytoskeleton, was more highly expressed and exhibited a wider distribution in the *siHDAC6* transfected group than the *siNC* transfected group (Figure 4C3,C4). LC3 was localized as puncta among ac-Tubulin (Figure 4C), and the LC3 puncta increased following HDAC6 knockdown (Figure 4C3,C4). These results indicated that HDAC6 regulated autophagic flux and ac-Tubulin.

**Figure 5:**
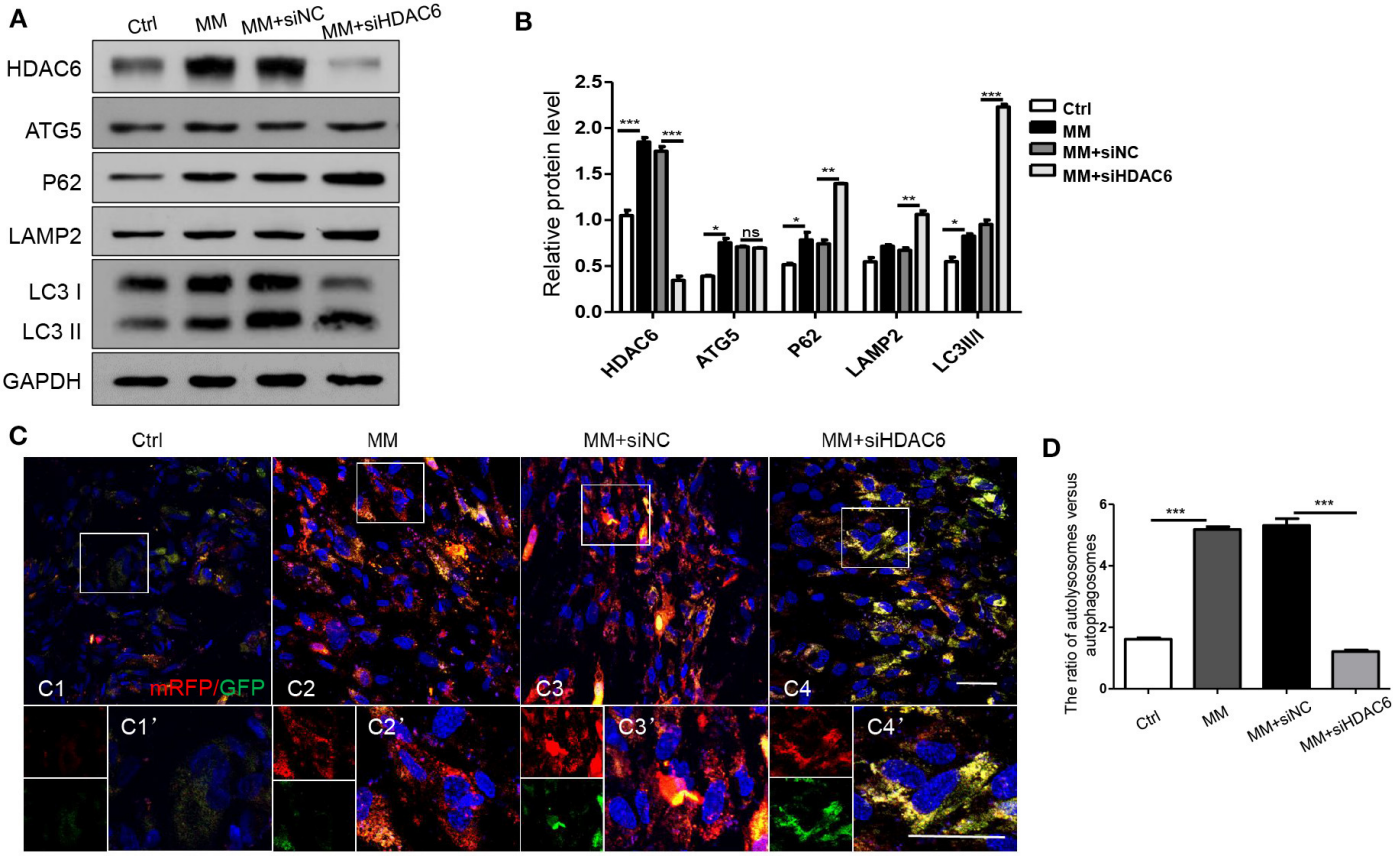
HDAC6 affects the fusion of autophagosomes and lysosomes during odontoblast differentiation. hDPCs were cultured in complete medium (Ctrl), mineralization-inducing medium (MM), MM following siNC (negative control) transfection, and MM following *siHDAC6* transfection. The expression levels of HDAC6, ATG5, P62, LAMP2, and LC3II/I were examined **(A)** and quantified using GAPDH as a loading control with Image J **(B)**. Results are representative of three independent experiments with similar results ± SD. Data were analyzed using one-way ANOVA with Tukey's multiple comparison test. ***P < *0.001*, **P < *0.01*, *P < *0.05*. **(C)** mRFP-GFP-LC3 plasmid was transfected into hDPCs prior to MM induction to trace autophagosomes and lysosomes. mRFP (red) indicated autolysosomes and yellow indicates autophagosomes. Scale bars =50 μm. **(D)** The ration of autolysosomes (sole red fluorescence without GFP) vs. autophagosomes (yellow fluorescence) was quantified by Image Pro Plus and analyzed using one-way ANOVA with Tukey's multiple comparison test. ***P < *0.001*.

To further confirm the specific stage at which HDAC6 is involved in autophagy, we detected autophagy during odontoblastic differentiation. We found the protein levels of HDAC6 and ATG5, and the ratio of LC3II/I increased when hDPCs were induced by MM (Figures 5A,B). This was consistent with their expression *in vivo*. Upon HDAC6 knocking down, ATG5 expression showed no obvious change, while P62 protein level increased, along with that of LAMP2 and the ratio of LC3II/I (Figures 5A,B). It suggested autophagsomes accumulation, and the decreased degradation. That illustrated the increased atuophagosomes was not because of increased autophagy induction, in contrast, it was due to autophagy process inhibition. Moreover, cells were transfected with mRFP-GFP-LC3 plasmid to trace autophagic flux. The GFP signal is sensitive to the acidic conditions of the lysosome lumen whereas mRFP is more stable. Therefore, colocalization of both GFP and mRFP fluorescence (yellow fluorescence) indicates autophagosmes have not fused with lysosomes, while a mRFP signal without GFP corresponds to autolysosomes (autophagosomes fused with lysosomes) (Klionsky et al., 2016). First, basic autophagy was observed in hDPCs without induction (Figure 5C1). Upon mineralization induction, mRFP fluorescent puncta increased, indicating that autophagy was increased and autophagic flux was occurring (Figures 5C2, **D**). When HDAC6 was knocked down, yellow fluorescent puncta increased in the cytoplasm, but red fluorescent (without GFP) puncta decreased (Figures 5C3,C4, **D**), indicating that autophagosomes were formed, but the fusion of autophagosomes and lysosomes was blocked, the autophagy activity was reduced. Ultimately, this indicated that knocking down of HDAC6 inhibited the fusion of autophagosomes and lysosomes and lead to decreased autophagy. These results indicated that HDAC6 regulated odontoblast differentiation by maintaining the fusion of autophagosomes and lysosomes through influencing the microtubule system within the cytoplasm.

## Discussion

HDAC6 is involved in a number of cellular processes due to its cytoplasmic localization and the various functions of its diverse substrates. HDAC6 is a component of the signaling pathway that regulates cell morphology and maturation (Destaing et al., 2005). Recently, HDAC6 has been shown to be involved in osteoblast differentiation in several osteoblastic cell lines (Ozaki et al., 2013; Wang et al., 2020) and valvular interstitial cells (Fu et al., 2019). However, there has been limited investigation of its role in odontoblast differentiation. Our study found that HDAC6 was more highly expressed in differentiated odontoblasts *in vivo*. HDAC6 expression also increased during odontoblast differentiation of hDPCs *in vitro*. Further, we found that knocking down HDAC6 in hDPCs impaired their odontoblastic differentiation ability. This illustrates that HDAC6 is involved in odontoblast differentiation.

Autophagy is important for proteins turnover and cell homeostasis, and participates in the maintenance of stem-like features and the remodeling of cells undergoing differentiation (Sotthibundhu et al., 2018). The role of autophagy in odontoblasts has recently been explored. Autophagy was detected in differentiated odontoblasts during embryonic and postnatal stages (Yang et al., 2013). In our studies, we found ATG5 and LC3 to be highly expressed in differentiated odontoblasts in incisors. Expression of LC3 and ATG5 also increased during odontoblast differentiation *in vitro*. Additionally, recent studies have also found the autophagic machinery to be involved in autophagy-dependent secretion of many proteins, such as TGF-beta, IL-1beta and some extracellular matrix components (New and Thomas, 2019). Many activities, such as protein transport, organelle recycling, and secretory activities, occur during odontoblast differentiation that are closely with autophagy activation. It is clear that autophagy is highly activated and required during odontoblast differentiation.

Recently, HDACs were found to regulate autophagy at multiple levels, such as regulating transcription factors to modulate autophagy, modifying the acetylation status and activity of autophagy proteins, and affecting cytoskeletal proteins to modulate the autophagy environment (Bánréti et al., 2013). We found that HDAC6 exhibited a similar expression pattern as autophagy molecules during odontoblast differentiation. HDAC6, ATG5 and LC3 were colocalized in differentiated odontoblasts in mouse incisors. The expression of HDAC6, ATG5, and LC3II increased during mineralization induction of hDPCs *in vitro*, suggesting that HDAC6 was closely associated with autophagy during odontoblast differentiation. Interestingly, LC3 puncta increased in the cytoplasm following HDAC6 knocking down in hDPCs. Additionally, following HDAC6 knocking down, ATG5 and Beclin1, proteins associated with the autophagy initiation stage, showed no change, while the autophagosome and lysosome markers LC3 and LAMP2 increased, along with autophagy substrate P62. This demonstrated that autophagic flux was affected by HDAC6 inhibition.

HDAC6 exhibits special characteristics when compared with other HDACs, beyond the canonical roles in epigenetic modification of histones and transcriptional activity. The deacetylation role of HDACs has been explored in cell differentiation. Our previous study showed that acetylation was involved in dentinogenesis, and HDAC3 regulated histone acetylation during odontoblast differentiation (Tao et al., 2019). HDAC6 could also deacetylate histones related to Runx2 (Ozaki et al., 2013) and acted as a co-repressor for Runx2 transcription to suppress the osteoblastic differentiation of MC3T3 cells (Zhu et al., 2011). However, recent studies have found that HDAC6 regulates the acetylation of some proteins, such as Cortactin and ac-Tubulin, and has weak histone deacetylase activity. HDAC6 deacetylates ac-Tubulin, which is involved in the transport of cargos along microtubule tracks (Boyault et al., 2007; Valenzuela-Fernández et al., 2008). Lee point out that HDAC6 could also promote autophagy by recruiting actin-remodeling machinery and assembling the F-actin network (Lee et al., 2010). In our study, the formation and distribution of ac-Tubulin increased after HDAC6 was inhibited with siRNA during odontoblast differentiation, LC3 puncta increased and localized along ac-Tubulin, suggesting that HDAC6 regulated microtubule and autophagosome transport during odontoblastic differentiation. Our results indicated that HDAC6 could regulate autophagy through the cytoskeleton and may be involved in autophagy machinery transport.

The role of autophagy depends on the autophagic flux, which includes the formation of autophagosomes in cells, their fusion with lysosomes, and then the degradation of cargos (Zhang et al., 2013). In our study, LC3II/I ratio up-regulated and LC3 puncta increased after HDAC6 was inhibited, which suggested autophagosomes accumulation. While, the autophagy initiation protein ATG5 and Beclin1 showed no obvious change, autophagy substrate P62 increased with HDAC6 inhibition. It illustrated increased autophagsosomes was not because of increased autophagy activation, but blocked autophagy flux. We also detected autophagic flux using the mRFP-GFP-LC3 plasmid, which enabled visualization of autophagic flux during odontoblast differentiation. When HDAC6 was inhibited, the number of autolysosomes was decreased but that of autophagosomes was increased. This suggested HDAC6 inhibition would block the fusion stage of autophagosome and lysosome, then lead to decreased autophagy activity. However Zheng et al. (2020b) found that HDAC6 inhibition restored autophagy flux in axonal regeneration and Li et al. (2019) found HDAC6 inhibition induced autophagy flux in BMSCs. As HDAC6 regulates autophagy at multiple levels, it influences autophagy differently due to the cell type and the environment. It facilitated the fusion of autophagosomes to lysosomes by promting F-actin remodeling in MEFs (Lee et al., 2010). It also promoted autophagosome-lysosome fusion and autophagy by deacetylating Cortactin in mouse embryonic fibroblasts (Wang et al., 2019). What is more, it showed that HDAC6 inhibition downregulated autophagy in differentiated breast cancer cells but upregulated autophagy in cancer stem-like cells (Sharif et al., 2019). It is complex of HDAC6 in regulating autophagy. So, as we found in our study, in odontoblasts, HDAC6 could keep autophagy process by maintaining the fusion between autophagosomes and lysosomes during odontoblast differentiation.

Further, odontoblasts are able to recognize the pathogen-associated molecular patterns released by bacteria from carious lesions and produce pro-inflammatory mediators in response (Farges et al., 2013). Odontoblast is important in inflammation defense. Our previous study also found autophagy played an important role in inflammatory defense in odontoblasts (Pei et al., 2016). As such, our study on the role of HDAC6 in regulating odontoblast differentiation and the relationship of HDAC6 and autophagy during odontoblast differentiation also provides a new perspective on the treatment of caries.

In conclusion, through investigating the role of HDAC6 in odontoblast differentiation and its relationship with autophagy during this process, we find that HDAC6 affects the fusion of autophagosomes and lysosomes to maintain autophagy flux, keep autophagy activity to involve in odontoblast differentiation. When HDAC6 is inhibited, autophagosomes can't fuse with lysosome, autophagy activity is decreased, which leads to down-regulation of odontoblastic differentiation capacity.

## Data Availability Statement

The original contributions generated for the study are included in the article/supplementary materials, further inquiries can be directed to the corresponding author/s.

## Ethics Statement

The studies involving human participants were reviewed and approved by the School and hospital of Stomatology, Wuhan University. Written informed consent to participate in this study was provided by the participants' legal guardian/next of kin. The animal study was reviewed and approved by Institutional Animal Care and Use Committees at the School and Hospital of Stomatology of Wuhan University.

## Author Contributions

YZ performed the research, collected and analyzed the data, and wrote the manuscript. HW and LZ performed the data collection and analysis. FP and ZC designed the research, supported financial support for the research, and revised the manuscript. All authors contributed to the article and approved the submitted version.

## Conflict of Interest

The authors declare that the research was conducted in the absence of any commercial or financial relationships that could be construed as a potential conflict of interest.
